# Bitter Taste Receptors for Asthma Therapeutics

**DOI:** 10.3389/fphys.2019.00884

**Published:** 2019-07-16

**Authors:** Ajay P. Nayak, Sushrut D. Shah, James V. Michael, Deepak A. Deshpande

**Affiliations:** Division of Pulmonary, Allergy and Critical Care Medicine, Center for Translational Medicine, Department of Medicine, Jane and Leonard Korman Respiratory Institute, Thomas Jefferson University, Philadelphia, PA, United States

**Keywords:** TAS2R, airway smooth muscle, gustducin, asthma, SCC, relaxation

## Abstract

Clinical management of asthma and chronic obstructive pulmonary disease (COPD) has primarily relied on the use of beta 2 adrenergic receptor agonists (bronchodilators) and corticosteroids, and more recently, monoclonal antibody therapies (biologics) targeting specific cytokines and their functions. Although these approaches provide relief from exacerbations, questions remain on their long-term efficacy and safety. Furthermore, current therapeutics do not address progressive airway remodeling (AR), a key pathological feature of severe obstructive lung disease. Strikingly, agonists of the bitter taste receptors (TAS2Rs) deliver robust bronchodilation, curtail allergen-induced inflammatory responses in the airways and regulate airway smooth muscle (ASM) cell proliferation and mitigate features of AR *in vitro* and in animal models. The scope of this review is to provide a comprehensive and systematic insight into our current understanding of TAS2Rs with an emphasis on the molecular events that ensue TAS2R activation in distinct airway cell types and expand on the pleiotropic effects of TAS2R targeting in mitigating various pathological features of obstructive lung diseases. Finally, we will discuss specific opportunities that could help the development of selective agonists for specific TAS2R subtypes in the treatment of asthma.

## Introduction

Gustatory chemosensory perception within the oral cavity of vertebrates allows for discernment of bitter, sweet, sour, salty, and umami (savory taste associated with monosodium glutamate) stimuli associated with ingested foods. This sensation is communicated either through non-receptor or receptor mediated mechanisms and is commonly described as “taste.” While salt and sour taste perceptions are hypothesized to be driven by epithelial sodium channel (ENaC) and acid-sensing ion channels (ASICs), respectively, (Heck et al., 1984; Kinnamon et al., 1988; Ugawa et al., 1998), sweet, umami and bitter sensations are mediated by oligomeric G protein-coupled receptor (GPCR) isoforms (Adler et al., 2000; Gilbertson et al., 2000; Sainz et al., 2001). Collectively, these chemoreceptors and taste associated-ion channels have been comprehensively studied on taste cells which are organized in taste buds within the four different gustatory papillae on the dorsal surface of the mammalian tongue. Chemoperception of taste is initiated by interaction of specific taste stimulus with the highly specialized cognate receptors on type II taste cells on the tongue and subsequent transmission of signals to the brain through various cranial nerves (Lee and Cohen, 2015). Evolutionarily, perception of taste (along with olfaction) has been considered to provide dietary advantage since it may indicate whether a particular food is corrupted and potentially harmful for consumption.

Studied predominantly within the oral cavity, surprisingly taste receptors have been recently described in extra-oral physiological systems (respiratory, gastrointestinal, nervous, circulatory, dermal, and hematopoietic), indicating that taste receptors have adaptive functions. Specifically, bitter taste receptors (TAS2Rs) have been identified in various extra-oral tissues, including gut, pancreas, testes, lungs, and bladder (Sternini et al., 2008; Deshpande et al., 2011; Clark et al., 2012; Xu et al., 2013; Wolfle et al., 2016). TAS2Rs impart distinct biological functions within these different tissue environments, emphasizing physiological layering and versatility. Most importantly for the scope of discussion here, the ectopic distribution of TAS2Rs in upper and lower respiratory structural (epithelial and airway smooth muscle cells) and immune cells has also been described recently (Deshpande et al., 2011; Clark et al., 2012; Lee and Cohen, 2015). In this review, we will discuss TAS2R biology in distinct cell types associated with the respiratory system, the specific signaling events that ensue TAS2R activation, and the physiological outcomes in cells (*in vitro*) and integrative model systems (*ex vivo* and *in vivo*) relevant to the pathology of obstructive lung diseases.

## TAS2Rs in Airway and Immune Cells: Subtypes and Physiological Functions

### TAS2R Expression in Distinct Airway Cell Types

In lungs, TAS2R expression has been confirmed in multiple airway cell types including airway smooth muscle (ASM) cells, distinct epithelial cell subtypes as well as resident (macrophages) and migratory hematopoietic (neutrophils, mast cells, lymphocytes) inflammatory cells (Shah et al., 2009; Deshpande et al., 2011; Maurer et al., 2015; Tran et al., 2018). In this section we will provide a brief overview of TAS2R expression in individual cell types.

#### Airway Smooth Muscle

In isolated ASM cells from healthy human donors, mRNA transcripts have been confirmed for multiple TAS2R subtypes (Deshpande et al., 2010). Transcripts of different TAS2Rs were confirmed in ASM tissues freshly dissected from human lungs. Specifically, transcripts of *TAS2R10*, *TAS2R14*, *TAS2R31* are highly expressed in donor ASM cells with 3 to 4-fold higher levels relative to *ADRB2* (which encodes for the β_2_ adrenergic receptor). Transcripts of other TAS2Rs such as *TAS2R5*, *TAS2R4*, and *TAS2R19* are also expressed at higher levels than *ADRB2*. In subsequent years, investigators have reported TAS2Rs in ASM isolated from guinea pigs and mice, thus providing useful *in vivo* and *ex vivo* systems to study the role of TAS2Rs in ASM cells (Pulkkinen et al., 2012; Zhang et al., 2013; Tan and Sanderson, 2014). Specifically, in these studies, *TAS2R4*, *TAS2R14*, and *TAS2R10* have been described in rodent ASM and TAS2R107 was shown in murine ASM. Immunofluorescence studies in both isolated ASM cells and tissue slices obtained from human and rodent lungs have confirmed the expression of different subtypes TAS2Rs at protein level. These findings suggest that the expression of TAS2Rs on ASM is evolutionarily conserved across multiple species. Of note, TAS2R subtypes are found in smooth muscle of other organs including vasculature, gastrointestinal tract and bladder and play a role in the physiological regulation of multiple organ systems (e.g., gastric emptying, vascular tone and bladder contractility) (Lund et al., 2013; Avau et al., 2015; Liu et al., 2017; Zhai et al., 2016).

#### Airway Epithelial Cells

**FIGURE 1:**
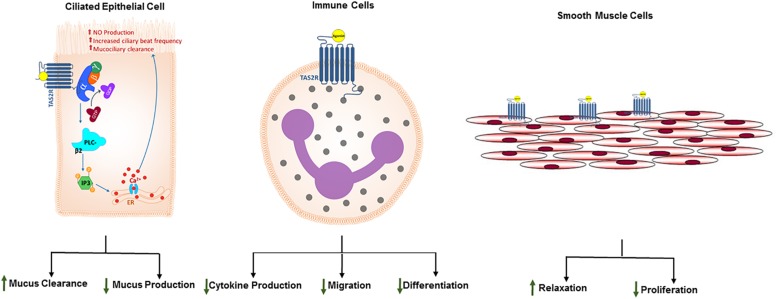
Expression of TAS2Rs and distinct functional outcomes of TAS2R agonism in airway cell types. Studies have demonstrated that different TAS2R subtypes are expressed on solitary chemosensory cells, ciliated epithelial cells, airway smooth muscle cells and immune cells. Proximal signaling events following TAS2R activation are invariant in taste cells and non-gustatory systems such as respiratory system. Moreover, these receptors have been evolutionarily engineered to adapt to the tissue environment. Consequently, TAS2R activation results in diverse functional outcomes depending on the cell type. While in taste cells, activation of TAS2Rs results in sensation of bitter taste, in epithelial cells and immune cells TAS2Rs play a crucial role in recognizing microbial products and mounting nuanced antimicrobial responses, specifically increased ciliary beat frequency, mucus clearance or antimicrobial nitric oxide generation. In ASM cells, TAS2Rs can be stimulated to induce ASM relaxation, bronchodilation, and inhibition of cellular proliferation. These functional outcomes of TAS2R activation in airway and immune cells prompt at potential therapeutic utility of the TAS2R agonism in obstructive lung diseases such as asthma.

Within the nasal cavity many cells that express TAS2Rs have been shown to be gustducin-positive and isolated cells have been confirmed for expression of TAS2Rs at transcript level (Finger et al., 2003; Shah et al., 2009; Wu et al., 2012; Figure 1). Specialized ciliated epithelial cells and solitary chemosensory cells (SCCs) express various TAS2R isoforms that are capable of recognizing specific chemical constituents within inhaled toxins and acyl-homoserine-lactones (AHLs) released by microbes. SCCs comprise 1% of total respiratory surface area (Workman et al., 2015) and express multiple TAS2R subtypes that are responsive to bitter tastants. SCCs, however, do not express TAS2R38, which is found on ciliated epithelial cells (Lee et al., 2012, 2014). For additional details on SCCs and their role in chemo-sensation and other physiological processes, the readers are referred to other reviews (Sbarbati and Osculati, 2003; Barham et al., 2013). In addition to SCCs, specialized chemosensory cells called *brush cells* (BCs) also express TAS2Rs (Saunders et al., 2013). TAS2Rs have also been shown to be expressed by motile cilia of the human airway epithelial cells (Shah et al., 2009). Gustducin-expressing epithelial cells are not limited to humans and have been reported to be scattered throughout the nasal epithelium in other species including rodents (Finger et al., 2003). Unlike ASM cells, expression of TAS2Rs on airway epithelium is restricted to specialized areas suggesting engagement of TAS2Rs in esoteric physiological and pathophysiological functions.

#### Immune Cells

In addition to structural cells of the lung tissue, TAS2Rs have also been identified in tissue resident and lung infiltrating immune cells (Orsmark-Pietras et al., 2013; Ekoff et al., 2014). TAS2Rs have been confirmed at transcript level using genome-wide expression analyses of isolated blood leukocytes. Furthermore, the expression of 3 TAS2Rs (13, 14, and 19) has been shown to be significantly increased in asthmatics (Orsmark-Pietras et al., 2013). In the same study, lymphocytes were demonstrated to have higher expression of many TAS2Rs relative to monocytes and neutrophils, with *TAS2R10* expression significantly upregulated in lymphocytic populations. Additionally, TAS2R38 has been reported in lymphocytes with high level of expression in CD4^+^ T lymphocyte populations, with extensive phenotyping revealing that the expression levels are higher in activated and memory T cell populations relative to naïve CD4^+^ T cells (Tran et al., 2018). In addition to lymphocytes, TAS2Rs have also been reported in monocytic and granulocytic innate immune cells (Maurer et al., 2015; Gaida et al., 2016). Of particular interest, *TAS2R38* expression has been reported in neutrophils by multiple investigators and has gained significant interest owing to its role in promoting antimicrobial action (Maurer et al., 2015; Gaida et al., 2016). Finally, nine distinct TAS2R subtypes have been reported in mast cells, with *TAS2Rs 4, 46*, and *14* expressed at high levels (Ekoff et al., 2014). Presence of TAS2Rs on immune cells opens a new avenue for therapeutic targeting in airway inflammatory diseases such as asthma (Figure 1).

### Functional Effects of Stimulation of TAS2Rs in Airway Cells

#### Airway Smooth Muscle

##### Relaxation

Activation of TAS2Rs on ASM cells results in robust relaxation (Figure 1), perhaps a serendipitous observation that attracted the attention of researchers from academia and pharmaceutical industry. The proximal signaling events that ensue TAS2R activation in ASM cells are marked by increased [Ca^2+^]_i_ in ASM cells. Initially, it was presumed that the spike in [Ca^2+^]_i_ in ASM cells following TAS2R activation by bitter taste compounds could result in ASM contraction (Deshpande et al., 2010). This is because, in cultured human ASM cells the magnitude of [Ca^2+^]_i_ transients evoked by bitter tastants is comparable to that of Gα_*q*_-coupled bronchoconstrictors, histamine, and bradykinin (Deshpande et al., 2010). Constituents of environmental pollutants could be presumably bitter tasting compounds and may engage TAS2Rs in airways and promote ASM contraction and consequently the physiological rejection of inhaled toxicants. Surprisingly, stimulation of TAS2Rs on ASM induces a relaxation response with substantial reversal of pharmacologically- induced contraction as demonstrated using isolated intact murine tracheal rings and human main-stem bronchi (Deshpande et al., 2010; Zhang et al., 2012, 2013). Further, microrheological techniques have demonstrated that the TAS2Rs expressed on isolated human ASM cells are primary targets that drive relaxation response on stimulation with bitter compounds (Deshpande et al., 2010). It is worth noting that TAS2R-mediated [Ca^2+^]_i_ elevation in ASM cells was not observed in freshly isolated murine ASM cells (Zhang et al., 2013) and in murine lung slices (Tan and Sanderson, 2014) contrary to what was seen in cultured human ASM cells. This discrepancy could stem from difference in the experimental model used in different studies. Furthermore, TAS2R activation in the presence of a contractile agonist results in inhibition of contractile agonist-induced elevation of [Ca^2+^]_i_ in ASM cells (Camoretti-Mercado et al., 2015).

Studies on TAS2Rs in ASM cells have shown that agonists of these receptors can reverse contraction induced by diverse contractile agonists. In our studies with murine tracheal tissue, we demonstrated that both quinine and chloroquine can reverse contraction induced by acetylcholine (ACh) and serotonin (Deshpande et al., 2010). Similarly, Belvisi et al. (2011) demonstrated that chloroquine can relax human airways contracted with histamine or carbachol. Airway relaxation following TAS2R agonism has since been unequivocally demonstrated by multiple groups in cells and tissues from mice (Deshpande et al., 2010; Zhang et al., 2013; Tan and Sanderson, 2014), humans (Belvisi et al., 2011; Grassin-Delyle et al., 2013) and guinea pigs (Pulkkinen et al., 2012) using isolated airways and lung slices. More recently, it was suggested that there is heterogeneity among TAS2R agonists in their ability to inhibit responses induced by distinct contractile agonists (Camoretti-Mercado et al., 2015). Specifically, chloroquine and denatonium demonstrate differential regulation of guinea pig ASM when pre-contracted with different contractile agonists (Pulkkinen et al., 2012). While denatonium (agonist for T2R4 and T2R10) preferentially inhibits cholinergic stimulation of guinea pig trachea, chloroquine (agonist for T2R3 and T2R10) can inhibit responses initiated by a broad spectrum of contractile agonists. Similar observations have been reported in human ASM cells where chloroquine and aristolochic acid (both bitter tastants) have been shown to differentially inhibit [Ca^2+^]_i_ elevation induced by histamine and endothelin (Camoretti-Mercado et al., 2015). Furthermore, in *in vivo* mouse model studies it was further demonstrated that TAS2R agonists reverse methacholine-induced bronchoconstriction (Deshpande et al., 2010). Collectively, these findings establish bitter tastants as novel bronchodilators.

##### Proliferation

In addition to being the main structural contributor of hypercontractile pathology in obstructive lung diseases, ASM cells can demonstrate a hyperplastic/hypertrophic phenotype that contributes to the airway remodeling in the lungs. This pathology is marked by an increase in fixed airway resistance (Nayak et al., 2018). In *in vitro* and *in vivo* (described in detail in later section) studies, agonists of the bitter taste receptor have demonstrated promising antiproliferative effects (Sharma et al., 2016). Our studies and a recent study from Liggett’s laboratory demonstrated that bitter taste receptor agonists such chloroquine, quinine and saccharin inhibit growth factor (PDGF)-induced ASM cell proliferation *in vitro* (Sharma et al., 2016; Kim et al., 2019). In murine model of asthma, studies further demonstrated mitigation of ASM cell proliferation by bitter tastants providing *in vivo* evidence for anti-mitogenic effect of TAS2R agonists (Sharma et al., 2017). Collectively, TAS2R agonist inhibit both ASM contraction and proliferation providing an opportunity to overcome two key features of asthma with TAS2R agonism.

#### Airway Epithelial Cells

Multiple epithelial cell types possess TAS2R and the key components of the signaling machinery that facilitate intracellular calcium signaling in response to stimulation by bitter agonists (i.e., gustducin, PLCβ_2_, TRPM5, and IP_3_R_3_) (Finger et al., 2003; Tizzano et al., 2006, 2011; Merigo et al., 2007; Gulbransen et al., 2008; Figure 1). Consistent with their ability to promote repulsive responses to noxious stimuli, TAS2Rs on upper airway (nasal) epithelial cells play a role in innate recognition and removal of harmful pathogens thus promoting immunity. This biological response to microbial secondary metabolites requires PLCβ2 and TRPM5 (Lee et al., 2014). SCCs regulate respiratory rate owing to their close proximity to parasympathetic nerve terminals and ability to release acetylcholine (ACh) (Krasteva et al., 2011, 2012). Stimulation of TAS2Rs on SCCs stimulates release of the neurotransmitter acetylcholine, which drives innate defenses by either eliciting a neurogenic inflammation or depressing respiration rate to limit continued inhalation of foreign insult (Finger et al., 2003; Tizzano et al., 2010; Krasteva et al., 2011, 2012; Saunders et al., 2014). In sinonasal epithelial cells, activation of TAS2R38 increases mucociliary clearance and directly kills bacteria suggesting bactericidal effect of TAS2Rs. Activation of TAS2Rs on human mucus secreting ciliated epithelial cells with bitter tastants results in an elevation of intracellular calcium, increased ciliary beat frequency and improved mucus clearance (Shah et al., 2009). TAS2Rs are located both extracellularly on the motile cilia and on the apical membrane of the cell. Receptor activation on ciliated epithelial cells may also result in induction of anti-microbial nitric oxide (Lee et al., 2012).

Within the nasal respiratory epithelium, chemosensory receptors are located on (1) a specialized population of trigeminal chemosensory cells that provide innate defense whereby inhaled toxic dusts or aerosols can trigger a physiological response which causes respiratory reflexes (Kinnamon et al., 1988; Lee et al., 2014), (2) SCCs that are uniquely positioned in close proximity to parasympathetic (vagus) nerve terminals (Ugawa et al., 1998; Krasteva et al., 2011, 2012; Saunders et al., 2013, 2014), (3) mucus producing ciliary epithelial cells that facilitate mucus clearance (Shah et al., 2009). Therefore, airway epithelial TAS2Rs play critical role in multitudes of airway pathologies. In the context of allergic airway diseases, certain microbes (such as molds) can be major sources of potent allergens that may exacerbate underlying asthma. Innate recognition of such toxic dusts and microbes could potentially help protecting the respiratory system from constant damage. Further, chemosensory cells in airway epithelium may also act as focal point of initiation and orchestration of allergen-induced type 2 immune response similar to what has been shown for tuft cells (chemosensory cells of gastrointestinal tract) in mediating parasite-induced Th2 immune response (Luo et al., 2019).

#### Immune Cells

Similar to epithelial cells, immune cells serve a critical function of maintaining homeostasis in tissue environments by neutralizing foreign insults. Immune cells are primarily armed with multiple cell surface receptors (TLRs and CLRs) and cytosolic proteins (cryopyrin or NALP3) that recognize specific pathogen associated molecular patterns (PAMPs). Immune cells also express TAS2Rs that further consolidate their ability to recognize microbial products and mount the appropriate response. In IgE-receptor–activated primary human mast cells, TAS2R agonists are found to inhibit the release of histamine and prostaglandin D2 (Ekoff et al., 2014). TAS2R agonists inhibit the release of multiple proinflammatory cytokines and eicosanoids in human blood leukocytes (Orsmark-Pietras et al., 2013). Bitter tastants (such as chloroquine, colchicine, erythromycin or ofloxacin) can inhibit cytokine release from tissue macrophages, myeloid-derived cell lines, circulating monocytes and monocyte-derived macrophages/dendritic cells (Jeong and Jue, 1997; Ianaro et al., 2000; Yasutomi et al., 2005; Jang et al., 2006; Ogino et al., 2009; Schierbeck et al., 2010; Vrancic et al., 2012). In addition to inhibiting cytokine production, our study in isolated human peripheral blood neutrophils suggest that activation of TAS2Rs results in inhibition of migration of immune cells (Sharma et al., 2017). Overall the studies noted above suggest that TAS2R agonism can inhibit cytokine/chemokine production and inflammatory cell chemotaxis resulting in a robust anti-inflammatory effect and promotion of immune tolerance. The molecular mechanisms underlying the anti-inflammatory effects of TAS2R activation need additional investigation.

### TAS2R – Receptor Organization, Core Signaling Events and Related Functional Outcomes

G protein-coupled receptors mediate signal transduction in all physiological systems and are activated by a wide range of endogenous ligands including chemicals, peptides and proteins. They are often critical for maintaining homeostatic functions in various tissue systems and consequently have been targeted for drug discovery in various diseases. Engagement of activating molecules (i.e., agonists) to the GPCR induces a conformational change in the receptor within the plasma membrane, allowing for binding of cognate heterotrimeric G proteins (G_q_, G_s_, G_i_ subunits) which initiates downstream signaling and accumulation of second messengers (e.g., Ca^2+^, cAMP) that culminates in distinct biological outcomes (Billington and Penn, 2003; Deshpande and Penn, 2006). Receptors of bitter taste sensation belong to the GPCR super-family of proteins and possess the typical structure of seven plasma membrane-spanning domains with a short extracellular N-terminal domain and an intracellular C-terminal domain. However, TAS2Rs differ from prototypical GPCRs in that TAS2Rs primarily associate with the cognate heterotrimeric G protein (gustducin) composed of Gα_gust_ and Gβγ subunits (Deshpande et al., 2010; Tizzano and Finger, 2013; Figure 1). Although gustducin expression is demonstrated in airway epithelial and smooth muscle cells, the functional role of Gα_gust_ in these non-gustatory cells remains unclear. Recently, it has been suggested that TAS2R signaling in ASM does not involve Gα_gust_ but instead ASM TAS2Rs couple to Gα_i_ subunits to induce airway relaxation (Kim et al., 2017). However, additional studies are needed to ascertain the role of Gα_gust_ or other Gα_i_ G proteins in airway relaxation and bronchodilation *ex vivo* and *in vivo* using knockout mice. It is also worth noting here that the Gα_gust_ is highly homologous to other members of the G_i_ family of G proteins.

Typically, ligand interaction with the receptor results in a conformational change of the receptor and subsequent association and then the dissociation of the G_α_ and G_β__bbb_ subunits. The conformation states of TAS2Rs with or without agonist activation is not well understood. Moreover, each of the TAS2R subtypes can be activated by multiple, chemically diverse group of agonists making the structure-activity relationship very complex. However, recent advances in computational analysis has provided hints at structural requirements of different domains on TAS2R for agonist binding (Born et al., 2013; Di Pizio and Niv, 2015; Di Pizio et al., 2016). Presumably, the disengaged G protein subunit mobilizes the membrane-associated phospholipase Cβ 2 (PLCβ2) enzyme, which in turn catalyzes the cleavage of the phospholipid phosphatidylinositol 4,5-bisphosphate (PIP_2_) into second messengers, inositol 1,4,5-triphosphate (IP_3_) and diacylglycerol (DAG) (Billington and Penn, 2003). Cytosolic interactions between IP_3_ and its receptor IP_3_R, an ion channel present on sarcoplasmic reticulum could result in elevation of [Ca^2+^]_i_. In taste cells, this signaling cascade results in elevation of intracellular calcium, opening of TRPM5 channel and in influx of Na^+^ causing depolarization at the cellular membrane resulting in release of ATP which acts as a neurotransmitter by activating purinergic receptors on nerves associated with taste buds. The resulting impulse is transmitted to the brain as bitter taste sensation (Finger et al., 2005; Chaudhari and Roper, 2010; Taruno et al., 2013; Peng et al., 2015). Absence of any of these signaling proteins, results in loss of sensation of bitter taste suggesting the necessary role of TAS2R-mediated signal transduction mechanism involving calcium. This signaling cascade is invariant and forms the core intracellular response in distinct cell types, however, the functional outcome is different and is tailored to the physiological environment wherein these receptors reside (Figure 1).

#### Mechanistic Basis of ASM Relaxation by TAS2Rs

**FIGURE 2:**
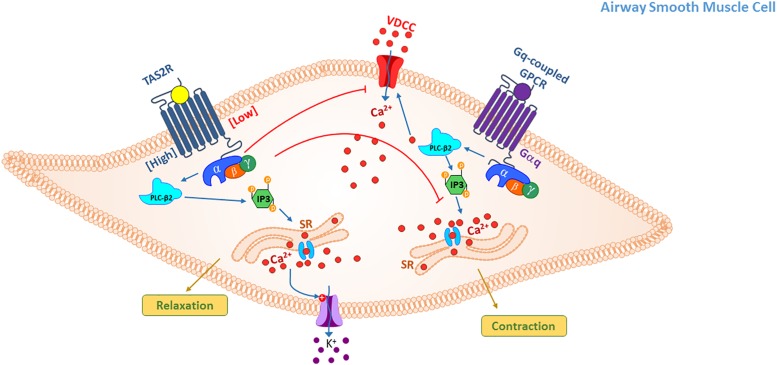
TAS2R signaling mediating relaxation in ASM cells. Activation of TAS2Rs on ASM cells results in relaxation, however, diversity in proximal signaling following receptor activation may occur with different agonists and agonist concentrations. Three basic mechanisms of action have been proposed: (1) elevation in intracellular Ca^2+^ from SR stores that in turn activate large conductance Ca^2+^ activated K^+^ (BK_Ca_) channels, alter membrane potential (hyperpolarize) and relax the smooth muscle; (2) Gβγ subunit-mediated inhibition of voltage-dependent calcium channel that are activated upon stimulation of ASM cells with contractile agonist thereby inhibiting calcium elevation and contraction; (3) inhibition of Ca^2+^ release from IP3 stores by Gq-coupled GPCR agonists thereby attenuating agonist-induced calcium elevation and contraction.

Independent research studies have consistently asserted that TAS2R agonism induces airway relaxation, however, the exact mechanism(s) by which this occurs is unclear (Figure 2). Detailed investigation into the mechanism of ASM relaxation induced by TAS2R activation suggests that unlike activation of β_2_ adrenergic receptor by receptor selective agonists, bitter tastants induce relaxation through mechanisms not dependent on generation of prototypical Gs-coupled signaling second messengers such as cAMP and activation of PKA, effects deemed essential for relaxation of ASM (Deshpande et al., 2010). Furthermore, neither epithelial cell generated nitric oxide nor PGE_2_ via activation of soluble guanylyl cyclase and prostaglandin receptors respectively, contributes toward TAS2R-mediated airway relaxation.

Findings from multiple groups suggest that TAS2Rs signaling in ASM cells involves complex interaction of diverse second messengers. Signaling via TAS2R in ASM is mediated via Gβγ subunit (contrary to other GPCRs in ASM) similar to what has been shown in taste cells (Deshpande et al., 2010). Further, TAS2R-induced ASM relaxation is dependent on activation of phospholipase C. However, the specific signaling downstream of PLC activation is somewhat controversial. In cultured ASM cells, our studies reveal elevation in intracellular calcium concentration by a variety of bitter tastants including chloroquine, quinine and saccharine (Deshpande et al., 2010). Although, the accumulation of [Ca^2+^]_i_ by bitter tastants and contractile agents such as histamine evoke differential functional outcomes. One leading hypothesis is that the stimulation by either agonist drives distinct spatiotemporal distribution of intracellular calcium. Stimulation with bitter tastants possibly compartmentalizes Ca^2+^ near the cell membrane resulting in opening of channels that collectively contribute to hyperpolarization at the cellular membrane (Deshpande et al., 2010). A similar mechanism has been described in neuronal cells where microdomains of concentrated [Ca^2+^]_i_ are associated with opening of BK_*Ca*_ channels (Fakler and Adelman, 2008). In ASM cells, activation of TAS2Rs and H_1_ histamine receptor by ligands (bitter tastants and histamine, respectively) results in generation of [Ca^2+^]_i_ signals within the cell. However, activation of TAS2Rs results in rapid and localized mobilization of [Ca^2+^]_i_ unlike response to histamine, which is predominantly diffused and delayed. However, in murine ASM cells and lung slices bitter tastants do not elicit calcium response but inhibit calcium elevation mediated by contractile agonists (Tan and Sanderson, 2014). Furthermore, a recent study revealed that modulation of calcium response in ASM is bitter tastant- and contractile agonist-dependent (Camoretti-Mercado et al., 2015). Calcium elevation, a rather atypical TAS2R signaling event appears to be heterogeneous and is restricted to specific combinations of a select few bitter tastants that may also activate/inhibit other heterotrimeric G_*q*_ or G_i_-coupled receptors. Currently, the specific factors that contribute to the compartmentalization of [Ca^2+^]_i_ and generation of possible microdomains remain unclear. Furthermore, the spatiotemporal dynamics of [Ca^2+^]_i_ following activation of TAS2Rs by distinct agonists remain ill-defined and is a subject of future research.

Studies have shown that downstream of TAS2R activation, large conductance Ca^2+^-activated potassium channels (BK_Ca_) are opened (Deshpande et al., 2010). BK_Ca_ channels that are gated by localized increase in calcium concentration and membrane potential are expressed on human ASM and are involved in regulating airway muscle tone (Morin et al., 2007; Martin et al., 2008). However, others have suggested that the activation of BK_Ca_ channels may not be required for relaxation by bitter tastants (Zhang et al., 2012). Again, these discrepancies could arise from experimental models and use of bitter tastants alone or in combination with contractile agonist. A study by Zhuge group suggested that TAS2R activation leads to Gβγ–dependent inhibition of voltage-gated calcium channels that are required for Gq-coupled GPCR-mediated calcium elevation and contraction (Zhang et al., 2012). Inhibition of contractile agonist-induced calcium elevation by TAS2R activation was also demonstrated by Sanderson group (Tan and Sanderson, 2014). This study specifically demonstrated that TAS2R activation leads to Gβγ–dependent inhibition of IP_3_-mediated calcium oscillations in murine lung slices. Study by Tan and Anderson has shown that TAS2R agonists dilate airways not by increasing [Ca^2+^]_i_ but through inhibition of IP_3_-mediated Ca^2+^ oscillations induced by contractile agonists of the G_*q*_-coupled receptors. This limits the pool of Ca^2+^ available to sustain the molecular signaling that contributes to contraction of ASM cells. Another study reaffirms findings by multiple investigators that TAS2Rs mediate ASM relaxation by unconventional mechanisms that are typical of bronchodilators currently in management of obstructive lung diseases. However, the findings from this group indicate that TAS2R agonists induce airway relaxation through inhibition of phosphatidylinositol-3 kinase (PI3K) (Grassin-Delyle et al., 2013). Specifically, inhibitors of PI3K (wortmannin, PI-828) potentiate the relaxing capabilities of bitter taste agonists. Elsewhere, it has been suggested that TAS2Rs regulate ASM function not by regulating pharmacomechanical coupling but by driving actin depolymerization and its subsequent dissociation from myosin (Tazzeo et al., 2012). While this mechanism has been specifically demonstrated for caffeine, it is unclear if this is a universal mechanism across all agonists.

Collectively, while multiple investigations described above have concluded that stimulation of TAS2Rs results in ASM relaxation, the specific mechanism/s by which this occurs has been controversial and has been debated extensively since the initial reporting of this phenomenon (Deshpande et al., 2010). The intrinsic heterogeneity within the TAS2R receptor-agonists combinations overlaid with species-specific variations in TAS2R expression may account for some of the differences. For instance, it was demonstrated that while agonist activity on TAS2R receptors increases [Ca^2+^]_i_, this phenomenon may not be universal for all bitter tasting compounds (Deshpande et al., 2010; Camoretti-Mercado et al., 2015). Furthermore, the authors also demonstrated that the choice of pre-contractile agonist could differentially alter intracellular Ca^2+^ management in ASM cells suggesting differential regulation of compartmentalized signaling in ASM cells. As a consequence of this compartmentalized spatiotemporal regulation of Ca^2+^, the membrane potential changes are distinct (likely involving certain channels). The study concluded that TAS2R activation results in activation of principally two distinct pathways based on their efficiency in managing cellular Ca^2+^. The pathway that induces Ca^2+^ accumulation is likely a less efficient pathway since the potency of agonists to achieve this response is high. Another second pathway likely competes with contractile agonists for the available Ca^2+^ pool, thus driving a competitive inhibition of contraction (Figure 2). The pharmacological heterogeneity is also further complicated by the application of a mechanical stress/force change on ASM tissue in many of the *ex vivo* physiological evaluations describing TAS2R function. Another aspect that could contribute to differences is due to potential off-target effects of TAS2R agonists that are not completely understood. One approach that has previously worked for refining our understanding of heterogeneity in GPCR signaling involves distinct E-prostanoid (EP) receptor subtypes that bind to the endogenous ligand prostaglandin E2 (PGE_2_) resulting in diverse signaling and functional outcomes. The development of EP receptor subtype-specific agonists has provided clarity on the physiological attributes endowed to each receptor (Michael et al., 2019). Additional efforts that engender the development of TAS2R receptor subtype specific agonists could potentially clarify the perplexing signaling differences noted in multiple studies.

Finally, one puzzling aspect of TAS2R physiology in airways is that while stimulation of TAS2Rs across all species stimulates airway relaxation, the potency and efficacy of the relaxant effect is different in mice (including different subspecies) compared to humans (Deshpande et al., 2010, 2011; Belvisi et al., 2011; Morice et al., 2011; An et al., 2012a; Zhang et al., 2012). Broadly, the use of cells/tissues from different species could potentially explain some of the differences across the studies. Studies on tissues/cells sourced from human donors are also often fraught with intrinsic variabilities. Furthermore, the distribution of specific TAS2R subtypes within the human bronchial tree is uncharacterized and could possibly account toward variations. Finally, methodological discordances in defining baseline contractile tone or resting passive tension could explain the inconsistencies reported in these findings. In future, additional studies with more refined receptor subtype-specific agonists in a unified experimental system are needed to ascertain the relative contribution of each signal transduction pathway.

#### Mechanistic Basis of Inhibition of ASM Proliferation by TAS2Rs

**FIGURE 3:**
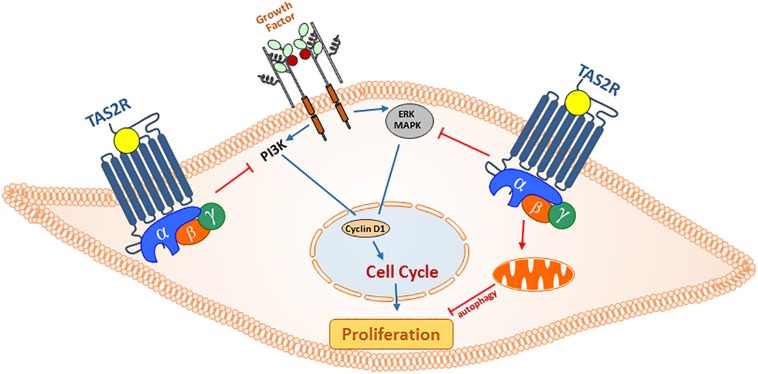
Anti-proliferative actions of TAS2R agonism in ASM cells. In asthma, secreted growth factors from infiltrating immune cells and resident epithelial cells can promote ASM cell hyperplasia through PI3 and MAP kinase pathways. Activation of TAS2Rs can regulate ASM cell proliferation by (1) exerting inhibitory action on cell cycle progression (ERK/MAPK and PI3K) and (2) promoting mitochondrial fragmentation and autophagy, and inhibiting cell survival.

As noted earlier, one promising feature of TAS2R agonists is their ability to regulate ASM proliferation, a significant advantage over current asthma medications. Multiple studies have clarified the signaling mechanisms that regulate ASM proliferation (Sharma et al., 2016; Pan et al., 2017). Stimulation with growth factors results in proliferative signaling which are orchestrated and sustained by activation of distinct pathways. Firstly, growth factors can activate their cognate tyrosine-kinase receptors and activate the mitogen-activated protein (MAP) kinase pathway that promotes cell proliferation. Furthermore, activated receptors can also recruit cytosolic phosphoinositide-3-kinase (PI3K) that phosphorylates phosphatidylinositol 4,5-bisphosphate (PIP_2_) to form phosphatidylinositol 3,4,5-triphosphate (PIP_3_), which is a second messenger that activates protein kinase B (Akt), which in turn can regulate activation of enzymes and transcription factors that promote cell growth (Figure 3). Agonists of TAS2Rs regulate ASM proliferation stimulated by growth factors by inhibiting specific checkpoints within these pro-growth pathways (Sharma et al., 2016). Specifically, TAS2R agonists (chloroquine and quinine) inhibit phosphorylation of Akt and p70-S6 kinase independent of PIP_3_ regulation, suggesting that it occurs downstream of PI3K. Furthermore, while TAS2R agonists do not inhibit acute activation p42/44 and p38 following stimulation of ASM cells with growth factors, they inhibit activation of the transcription factors AP-1 (Activator protein-1), and E2F with an associated decrease in expression of cell cycle genes including cyclin D1, which are essential for cell cycle progression (Figure 3). Collectively, these findings demonstrate significant capability of TAS2R agonists in regulating ASM cell proliferation. A recent study by Kim et al. (2019) showed that TAS2R agonists inhibit cell proliferation in ASM cells obtained from healthy and asthmatic donors, and TAS2R agonist-induced antimitogenic effect on ASM involves inhibition of ERK MAP kinase activity in a time-dependent manner (Figure 3). The authors also demonstrate that different structurally diverse TAS2R agonists inhibit ASM proliferation to a different degree suggesting multiple mechanisms may contribute to the anti-mitogenic effect of TAS2R agonists (Kim et al., 2019).

More recently, we have shown that in addition to arresting cell cycle progression, bitter tastants can also alter mitochondrial function and induce autophagy in ASM cells (Pan et al., 2017). Specifically, using a combination of ultrastructural analysis and biochemical approaches we demonstrate that TAS2R agonists stimulate formation of autophagosomes to inhibit cell survival (Figure 3). Furthermore, TAS2R agonists not only decrease mitochondrial membrane potential and increase ROS generation, they drive physical changes in mitochondrial structure by inducing fragmentation. Activation of TAS2Rs increased expression of Bnip3 and subsequently increased localization of dynamin-like protein 1 (DLP-1) to mitochondria with concurrent mitochondrial fragmentation and organelle dysfunction. These findings demonstrate that the activation of TAS2Rs induces cell death through diverse mechanisms, thus making them promising targets for development of anti-asthma therapy.

#### Epithelial Cell Function Modulation by TAS2R Agonists

As noted earlier, the signaling events upon TAS2R activation are invariant in distinct cell types and results in mobilization of intracellular Ca^2+^ from the intracellular stores. However, there is considerable diversity in specific events downstream of Ca^2+^ accumulation within each cell type that contributes toward innate defense. For e.g., following stimulation by bitter tastants, sinonasal epithelial cells increase production of nitric oxide (NO) which can be toxic to invading pathogens (Lee et al., 2014). In particular, T2R38 has been shown to directly recognize *N*-acyl homoserine lactones (AHL) (a bitter tasting compound) from gram-negative bacteria (*Pseudomonas aeruginosa*) (Lee et al., 2012). In gram-negative bacteria and certain molds, accumulation of AHL serves as a quorum sensing strategy to regulate biofilm formation which is an assemblage of microbial cells that is enclosed within a polysaccharide matrix, and are an important sue toward controlling establishment of infectious diseases (Donlan, 2002; Taghadosi et al., 2015). T2R38-AHL interaction yields a Ca^2+^-dependent nitric oxide production, increased ciliary beat frequency, and increased mucociliary clearance of inhaled pathogens. Therefore, the airway epithelium TAS2R-mediated detection of AHL has an important role in imparting immunity in the airways, (Tizzano et al., 2010) in which TAS2Rs serve as pattern recognition receptors (PRRs) capable of directly engaging the molecules secreted by pathogens. While activation of TAS2R38 on ciliated epithelial cells generates bactericidal nitric oxide (Lee et al., 2012, 2014), stimulation of TAS2Rs on SCCs stimulates release the neurotransmitter acetylcholine, which drives innate defenses by either eliciting a neurogenic inflammation or depressing respiration rate to limit continued inhalation of foreign insult (Finger et al., 2003; Tizzano et al., 2010; Krasteva et al., 2011, 2012; Saunders et al., 2014). SCCs also demonstrate unique cooperative capabilities with adjacent cells to enhance innate immunity. Activation of TAS2Rs on SCCs stimulates a Ca^2+^ wave within the cell, which propagates into adjoining cells through gap junctions and elicit release of antimicrobial peptides and β-defensins (Bezencon et al., 2008). In ciliated epithelial cells, TAS2Rs are located both extracellularly on the motile cilia and on the apical membrane of the cell, and receptor activation induces an increase in intracellular Ca^2+^, leading to increased ciliary beat frequency, possibly through induction of nitric oxide and cGMP-protein kinase G pathway (Salathe, 2007; Figure 1).

As we develop a better understanding of signaling in distinct airway epithelial cell types in normal and diseased state, and other factors that could serve as modulators of TAS2Rs signaling, we may be able to tease out the functional role for specific TAS2R subtypes and tailor specific agonists for desired physiological outcomes (Table 1).

**TABLE 1:** TAS2R subtypes and broad selectivity of receptor agonists.

**Bitter tastants**	**Human receptor**	**Murine receptor**
Denatonium	TAS2R4, TAS2R10, TAS2R44	TAS2R108
6-n-propyl-2-thiouracil (PROP)	TAS2R4, TAS2R38, TAS2R51, TAS2R61	TAS2R108
Dextromethorphan	TAS2R10	
β-glucopyranosides	TAS2R16	
Chloroquine	TAS2R10, TAS2R3, TAS2R39, TAS2R7	
Acyl-homoserine lactones	TAS2R38	
Quinine	TAS2R10, TAS2R14, TAS2R4, TAS2R46, TAS2R39, TAS2R31, TAS2R7, TAS2R40	
Colchicine	TAS2R4	
Strychnine	TAS2R10, TAS2R46	
Noscapine	TAS2R14	
Saccharin	TAS2R31	
Cycloheximide		TAS2R5
Lidocaine		TAS2R5
Picrotoxinin	TAS2R14	

## Role of TAS2Rs in Asthma: Findings From Animal Models and Human Cells and Tissues

### Animal Studies

Since their discovery on ASM cells, significant interest has been stimulated in gaining mechanistic insights into TAS2R signaling and their biology with an aim to regulate ASM function for therapeutic relief of obstructive lung diseases. Asthma is the leading cause of respiratory morbidity and increasing financial burden especially in developed countries. The disease has reached epidemic proportions affecting >330 million people worldwide, with over >250,000 deaths annually. Respiratory distress is primarily driven by airflow obstruction in the conducting airways resulting from constriction of ASM of the bronchi, excessive mucus production and airway inflammation orchestrated by cellular and secreted mediators. Clinical presentations can be managed with variable degree of success using therapeutic options available currently. However, the chronic pathological state in the lungs drives tissue remodeling (extensive structural changes to lung tissue), which is challenging to reverse with modern clinical interventions. Consequently, more than half the patients affected by asthma (and COPD) exhibit inadequate control of the disease with current therapeutic regimens (Kraan et al., 1985; Cheung et al., 1992). TAS2Rs have emerged as promising novel therapeutic targets owing to their efficacious bronchodilatory effect.

In the lung, the ASM cells express a repertoire of GPCRs that when activated modulate airway functions (contractile, proliferative and synthetic) (Deshpande and Penn, 2006). The discovery of TAS2Rs in structural and immune cells that drive asthma pathology provides an opportunity to mitigate the disease pathology through development of novel agonists that target these receptors. The unique physiological properties of TAS2Rs described here and elsewhere underline their clinical relevance (Gerthoffer et al., 2013; Liggett, 2013; Pera and Penn, 2016). Indeed, in severe asthmatics, upregulation of TAS2R sub-types on peripheral blood leucocytes has been established, underscoring their pharmacological accessibility as novel therapeutic targets for asthma (Orsmark-Pietras et al., 2013). Furthermore, in asthmatic ASM cells and lung tissue slices, neither the expression nor the signaling and function of TAS2Rs is impaired under inflammatory conditions (An et al., 2012b; Robinett et al., 2014). More importantly, TAS2R signal-function outcomes are not hindered by β2-adrenergic receptor tachyphylaxis (An et al., 2012b). This is very important because one of the challenges of using beta agonists is tachyphylaxis due to compromised functions of β_2_ adrenergic receptors (Beasley et al., 1999; Salpeter et al., 2010; Walker et al., 2011). In fact, in murine model, TAS2R agonists inhibit bronchoconstriction induced by methacholine efficaciously despite a modest bronchodilation by beta agonist (Deshpande et al., 2010). Furthermore, in the original article characterizing TAS2Rs on murine airway smooth muscle cells, a greater efficacy was demonstrated for bitter tastants in evoking bronchorelaxation compared to β_2_ agonists, thus making them appealing therapeutic candidates for the management of airway diseases (Deshpande et al., 2010).

In pre-clinical models of allergen-induced (house dust mite and ovalbumin) asthma, inhalation of bitter compounds grant bronchoprotection and mitigate allergen-induced airway inflammation (Deshpande et al., 2010; Sharma et al., 2017). Treating mice with bitter taste compounds *via* inhalation of drug aerosols or intranasal administration results in ablation of most cardinal features of allergen-induced asthma (Sharma et al., 2017).

Treatment with chloroquine and quinine also has a profound effect on infiltration by inflammatory cells in the airways and within the lung parenchyma. For many years, corticosteroids (inhaled and systemic) have been mainstay for management of bronchial inflammation in asthmatics. In addition to being a viable candidate for ASM relaxation, bitter compounds may be potentially employed in controlling airway inflammation. TAS2Rs have been reported on diverse human leukocyte populations, including circulating granulocytes, T lymphocytes and monocytes that are all central to asthma pathology (Malki et al., 2015). Microarray methods to analyze global changes in gene expression in leukocytes isolated from severe asthmatics revealed significant upregulation of genes for various TAS2R subtypes (Orsmark-Pietras et al., 2013). Expression of multiple TAS2R transcripts is relatively higher in lymphocytes compared to monocytes or granulocytes, consistent with another study involving healthy donors (Malki et al., 2015). There is also a significant shift in expression of TAS2R subtypes among healthy donors and asthmatics. For instance, in healthy donors, *TAS2R31* transcripts are reportedly most abundant in isolated circulating cells. Further, while *TAS2R10* expression is highest in leukocytes from severe asthmatics, its expression is lowest among TAS2Rs evaluated in leukocytes isolated from healthy donors, indicating regulation of receptor expression in disease states. Furthermore, *TAS2R5* expression in leukocytes from asthmatics demonstrates significant correlation with bronchial hyperresponsiveness to methacholine challenge. In the same study, treatment of leukocytes from whole blood co-stimulated with LPS and chloroquine or denatonium resulted in significant inhibition of production of allergic cytokines IL-4, IL-5 and IL-13. However, few have investigated the precise mechanism by which TAS2R agonists regulate immune cell function. Bitter compounds may also limit airway inflammation by regulating chemotaxis of immune cells and consequently their recruitment to airways (Sharma et al., 2017). Inhibition of neutrophil recruitment by TAS2R agonism could potentially benefit in cases of neutrophilic severe asthma or in mitigating COPD-associated exacerbations. In both cases patients respond poorly to inhaled corticosteroids possibly due to lower expression of glucocorticoid receptors in infiltrating neutrophils (Plumb et al., 2012).

Another proposed mechanism by which TAS2R agonists may regulate airway inflammation is by inhibiting IgE receptor crosslinking-dependent activation of mast cells (MCs) (Ekoff et al., 2014). Activated MCs are recruited to the ASM in asthmatics and contribute to airway hyperresponsiveness (Brightling et al., 2002). Airway infiltrating mast cells influence ASM function by secreting pro-contractile and proliferative mediators such as histamines, leukotrienes, tryptase, TGF-β etc., (Page et al., 2001). Human mast cells isolated from cord blood express multiple TAS2Rs with *TAS2R4* in relatively higher abundance (Ekoff et al., 2014). Brief treatment of isolated mast cells with bitter compounds (chloroquine, denatonium) prior to receptor crosslinking and subsequent activation significantly inhibits release of histamine and PGD_2_ indicating a different mechanism of directly limiting exposure of ASM to pro-contractile agents. Finally, if epithelial TAS2R subtypes regulate allergen-induced airway inflammation similar to what has been shown in parasite-induced Th2 immune response by TAS2Rs expressed on tuft cells in intestinal tract is not known.

Along with airway inflammation, airway remodeling forms a crucial hallmark of asthma and chronic obstructive lung diseases. Remodeling is marked by the increased thickening of ASM resulting from underlying cellular proliferation. Remodeling causes conducting airways to be refractory to bronchodilatory treatments and current treatment regimens have suboptimal effect on airway remodeling (Bonacci and Stewart, 2006; Halwani et al., 2010). At clinical level, there is no evidence of significant inhibitory effects by β agonists, the current gold-standard of bronchorelaxation on airway remodeling (Halwani et al., 2010). Bronchial epithelial cells from severe asthmatics release paracrine factors such as leukotrienes that can regulate ASM proliferation (Trian et al., 2015) and although antagonists of CysLTR (montelukast) demonstrate significant anti-mitogenic effects *in vitro* and *in vivo*; no studies in humans have supported these findings (Halwani et al., 2010; Kelly et al., 2010). Thus, the therapeutic mitigation of remodeling has emerged as the “holy grail” of modern-day asthma therapy, and TAS2Rs provide a golden opportunity for anti-asthma drug development.

*In vitro* studies indicate that TAS2R agonists curtail growth factor-stimulated cell proliferation in ASM cells obtained from healthy and asthmatic donors alike in a dose-dependent manner (Sharma et al., 2016; Kim et al., 2019). Mechanistically, TAS2R agonists inhibit ASM proliferation by multiple mechanisms: (1) inhibiting the growth factor-activated protein kinase B (Akt) phosphorylation, (2) inhibiting transcription factors AP-1, STAT3, E2 factor and NFAT, and (3) inhibiting genes associated with cell cycle progression. The anti-mitogenic activity of TAS2R stimulation is independent of PKA activation, which is partly responsible for similar functions for β_2_AR (inhibition of ∼25%) and PGE_2_ (inhibition of ∼75%) (Kong et al., 2008; Yan et al., 2011; Sharma et al., 2016). The anti-mitogenic effects of TAS2R agonists also translate in pre-clinical asthma model. Using a chronic allergen challenge model, where pathological features of remodeling can be studied extensively, treatment with bitter taste compounds (chloroquine and quinine) reverses cardinal features of airway remodeling (Sharma et al., 2017). Specifically, treatment with bitter compounds inhibited the expression of calponin, smooth muscle α-actin, and smooth muscle myosin heavy chain. Furthermore, levels of MMP-8 (neutrophil collagenase), pro-MMP-9 (gelatinase) and MMP-12 (macrophage metalloelastase) were significantly lower in the tissues following treatment with TAS2R agonists. Collectively, these *in vitro* and *in vivo* studies suggest that TAS2R agonism mitigates features of fibrotic airway remodeling in asthma.

Treatment with bitter tastants also limits mucus secretion and goblet cell hyperplasia. As described above, TAS2Rs found on ciliary epithelial cells control ciliary beat frequency and mucus clearance. Recent studies suggest that chronic use of beta agonists promote mucus production potentially contributing to asthma severity (Nguyen et al., 2017). TAS2R-mediated inhibition of mucus accumulation favors therapeutic utility of TAS2Rs in asthma. In prophylactic as well as the treatment model, mice treated with chloroquine or quinine exhibit significant reduction in AHR following methacholine challenge (Sharma et al., 2017). Interestingly, following 3 weeks of treatment with chloroquine and quinine, the bronchodilatory effect of TAS2R agonists was not diminished in both PBS and HDM challenged animals. These data suggest that treatment with TAS2R does not lead to functional tachyphylaxis.

Collectively, these studies underline the multimodal and pleiotropic manner in which TAS2Rs can be targeted for asthma relief and underline the value of developing TAS2Rs as novel targets for asthma treatment either alone or as an accessory to current regimens (Figure 1).

## Receptor Polymorphisms, Disease Association and Development of Personalized Medicine-Based Approaches

To date, over 40 TAS2R genes have been discovered in humans and mice that encode for proteins of ∼ 300-330 amino acids in length (Bachmanov and Beauchamp, 2007; Deshpande et al., 2010). Genes coding for TAS2R subtypes are distributed on chromosomes 5, 7, and 12 in humans and chromosomes 2, 6, and 15 in mice. TAS2Rs constitute of 25 subtypes that share 30–70% of their amino acid sequence homology (Behrens and Meyerhof, 2006). Airway cells express multiple subtypes of TAS2Rs and up-regulation of TAS2R gene expression has been reported in leukocytes of subjects with severe asthma (Pulkkinen et al., 2012). These studies demonstrate that TAS2Rs contribute to physiological and pathological phenotypes and that TAS2R genetic variations may contribute to disease severity and risk. The next critical step in TAS2R research is to determine which specific TAS2R subtypes are most important with respect to asthma pathogenesis and potential anti-asthma therapy. Given the strong evidence that TAS2Rs are involved in ASM relaxation and ASM proliferation it is logical to assume that one or more TAS2R genes regulate airway functions. In fact, recent studies have suggested functional *bias* among different TAS2R agonists (Camoretti-Mercado et al., 2015). To further advance this field of TAS2R research, it is imperative that we determine specifically which TAS2Rs affect asthma phenotype.

Humans differ in perception of bitter taste which is attributed to single nucleotide polymorphisms (SNPs) in TAS2R genes (Table 2; Chamoun et al., 2018). Substantial literature has been dedicated toward understanding the functional impact of polymorphisms associated with the *T2R38* gene owing to differential ability in taste perception of the bitter compound phenylthiocarbamide (PTC). Three SNPs (at positions 49, 262, and 296) of the *TAS2R38* gene form two common haplotypes- either PAV (proline, alanine and valine) or AVI (alanine, valine, and isoleucine). The PAV haplotype is considered a “taster,” while inheriting the AVI haplotype is considered a “non-taster” of bitter taste sensation (Drayna, 2005). Aside from taste preference, TAS2R SNPs may contribute to a range of pathologies. For instance, the *TAS2R38* PAV haplotype is correlated with a non-smoking preference, while the AVI (non-taster) haplotype is more common among smokers (Risso et al., 2016). Thus, possessing a specific TAS2R SNP distribution may be beneficial for aversion of unhealthy habits such as smoking although the current literature indicates conflicting observations which limits generalization (Mangold et al., 2008; Keller et al., 2013; Aoki et al., 2014).

**TABLE 2:** Taste receptor expressed on ASMs (order of level of expression) and their genetic variants.

**Genes**	**Amino acid variations in protein**
TAS2R10	T156M
TAS2R14	T86A, I118V, R174STOP, L201F, K225K
TAS2R31	R35W, M162L, Q217E, A227V, V240I
TAS2R5	G20S, S261?, P113L, Y167C, R209T, R213Q, R294L
TAS2R4	R3Q, Y6S, F7S, F62L, T74M, V96L, S171N, I191V
TAS2R19	F15S, V32I, C106Y, K126Q, R299C
TAS2R3	–
TAS2R20	S75V, K79E, H143Q, H148N, I236V, F252S, R225L
TAS2R45	C76Y, V132M, Q210H, R298T
TAS2R50	S125N, C203Y
TAS2R30	L235F, C238R, C250L, F252L, L281W, P307H
TAS2R9	S104T, K170Q, A187V, L238V, L304F, P309L
TAS2R13	H94R, N149S, N259S
TAS2R42	Y175F, L187L, F196S, G255W, Y265C
TAS2R46	F36V, S201S, L228M, W250STOP, S309P
TAS2R1	P43L, R111H, C141, R206Y
TAS2R8	E16K, M308V

*Sourced from GPCR natural variant database (http://nava.liacs.nl) and NCBI SNP database (dbSNP) (https://www.ncbi.nlm.nih.gov/snp).*

The extent to which polymorphisms of individual TAS2Rs impact the functionality of cells associated with the respiratory system is poorly understood. Polymorphisms in TAS2Rs have been linked to chronic rhinosinusitis (CRS), which is an inflammatory disorder of the nose and paranasal sinuses and in asthma (Adappa et al., 2013; Gallo et al., 2016; Yoon et al., 2016). Some have shown that the *rs10246939* SNP (*TAS2R38* nucleotide 296 to valine) and other TAS2R polymorphisms are associated with CRS (Adappa et al., 2013; Mfuna et al., 2014; Cohen, 2017). However, others have demonstrated a lack of an association of haplotypes between CRS patients, either with (CRSwNP) or without (CRSsNP) nasal polyps, (Gallo et al., 2016). However, at least one study strongly supports identification of polymorphisms in TAS2Rs as possible genetic markers to predict outcomes in asthma (Yoon et al., 2016). Although these association studies have identified a possible link between TAS2R polymorphisms and respiratory diseases, a causal link is yet to be established and the specific contribution toward physiological process remains to be defined.

## Current Limitations and Future Opportunities

Advances in our understanding of the underlying mechanisms that result in airway relaxation and antimitogenic effect following activation of TAS2Rs has been fraught with few challenges. The expression of TAS2Rs and relaxant effect of TAS2R agonists is species dependent (Deshpande et al., 2011). Furthermore, between mice and humans, some TAS2Rs lack genetic or pharmacological homologs, making it difficult to interpret the data from different species. *In vitro* and *in vivo* studies have also identified bitter tasting ligands for most human, but not murine TAS2Rs (Table 1; Chandrashekar et al., 2000; Bachmanov and Beauchamp, 2007; Meyerhof et al., 2010). Moreover, receptor specific knockout models are not practical considering multiple receptor subtypes can interact with a given ligand. Furthermore, expansive studies in human tissues and human subjects are challenging and lack of specific reagents (e.g., receptor-specific agonists and antagonists) to study TAS2R can all further complicate interpretation.

In the context of obstructive airways diseases, TAS2Rs clearly present promise of therapeutic potential due to their pleiotropic effects on multiple cell types in the lungs that collectively orchestrate distinct pathological features of the disease state. Unfortunately, currently available agonists of TAS2Rs demonstrate poor affinity to their targets and exhibit substantial promiscuity in activation of TAS2R subtypes. The number of compounds that trigger bitter taste sensation are significantly in excess to the number of *TAS2R* gene-encoded proteins, thus emphasizing promiscuity in receptor activation (Behrens et al., 2004; Di Pizio and Niv, 2015). It is possible that evolutionarily TAS2Rs have been adapted to be low-affinity receptors to allow for broad tuning of receptor activation by heterogeneous insults (Born et al., 2013). Nevertheless, gaining a better understanding of the structural details of refined agonists with higher affinity and receptor subtype selectivity will assist in clarifying the role of individual receptor subtypes on cell populations critical to asthma pathology. Development of TAS2R subtype selective agonists could be crucial in resolving some of these issues but has been hindered primarily due to unresolved crystal structure of a prototypical receptor. Discovery of TAS2R agonism in extra-oral tissue systems has generated significant interest in developing small molecules (agonists and antagonists) with improved receptor specificity and high-affinity. To this effect, traditional medicinal chemistry-based approaches as well as computational modeling have gained momentum (Di Pizio and Niv, 2015; Di Pizio et al., 2016; Karaman et al., 2016). Recently, significant strides have been made in exploiting the unique structural features of TAS2Rs that enable binding to diverse group of synthetic and naturally occurring chemical compounds (Di Pizio and Niv, 2015; Di Pizio et al., 2016; Karaman et al., 2016). Specifically, TAS2R promiscuity is owing to a large binding pocket and this structural disposition could possibly allow for engineering of novel agonists with modifications that could refine our ability to “fine tune” a “broadly tuned” receptor. These and other computation approaches may provide additional insight into TAS2R pharmacology and propel rational drug design.

## Conclusion

Expression and functional role of TAS2Rs on immune cells, epithelial cells and ASM suggest that TAS2Rs may be potential targets for improving bronchodilatory function and resolving immunological dysregulation and remodeling changes in individuals with obstructive lung diseases. From a clinical perspective, the perceived superior efficacy of some of the bitter tastants, lack of receptor tachyphylaxis, and availability of a large pool of compounds with established pharmacokinetic and pharmacodynamics properties are all attractive (perhaps promising) features of TAS2Rs. TAS2R-mediated bronchodilatory effect is additive to beta agonist-induced bronchodilation suggesting potential use of bitter tastants as adjuvant therapies. Therefore, TAS2Rs are attractive candidates for multimodal treatment of obstructive lung diseases either alone or in combination with current therapeutic regimens. Research into TAS2R agonists with increased specificity to receptor subtypes will aid in providing clarity to TAS2R physiology.

## Author Contributions

All authors listed have made a substantial, direct and intellectual contribution to the work, and approved it for publication.

## Conflict of Interest Statement

The authors declare that the research was conducted in the absence of any commercial or financial relationships that could be construed as a potential conflict of interest.
